# Late presentation of a cauda equina lesion with a vulval abscess: a case report

**DOI:** 10.1186/s13256-021-03012-z

**Published:** 2021-09-14

**Authors:** Chanil Deshan Ekanayake, Deepal Weerasekera, Dilini Dissanayake, Ranga Wickramarachchi, Saman Pushpakumara, Dumitha Govindapala

**Affiliations:** 1 Department of Clinical Sciences, Faculty of Medicine, General Sir John Kotelawala Defence University, Colombo, Sri Lanka; 2 University Hospital-General Sir John Kotelawala Defence University, Colombo, Sri Lanka

**Keywords:** Cauda equina syndrome, Vulval abscess, Saddle sensory loss, Bilateral sciatica, Perineal hypoesthesia, Urinary retention

## Abstract

**Background:**

Cauda equina syndrome is a rare clinical condition that requires prompt diagnosis and timely surgical decompression with postoperative rehabilitation to prevent devastating complications.

**Case presentation:**

A 55-year-old Sinhalese woman presented with a vulval abscess, with a history of involuntary leakage of urine for the last 7 years. Her sexual activity has been compromised due to coital incontinence, and she had also been treated for recurrent urinary tract infections during the last 7 years. On examination, a distended bladder was found. Neurological examination revealed a saddle sensory loss of S2–S4 dermatomes. There was no sensory loss over the lower limbs. Bladder sensation was absent, but there was some degree of anal sphincter tone. Motor functions and reflexes were normal in the limbs. Magnetic resonance imaging revealed L5–S1 spondylolisthesis. Ultrasound imaging confirmed the finding of a distended bladder, in addition to bilateral hydroureters with hydronephrosis. An incision and drainage with concomitant intravenous antibiotics were started for the vulval abscess. An indwelling catheter was placed to decompress the bladder and to reduce vulval excoriations due to urine. Bilateral ureteric stenting was performed later for persistent hydronephrosis and hydroureter despite an empty bladder.

**Conclusion:**

This is a tragic case that illustrates the devastating long-term sequelae that ensues if cauda equina syndrome is left undiagnosed. It reiterates the importance of prompt referral and surgical decompression.

## Background

Cauda equina syndrome (CES) is a rare condition with an incidence of 1 in 100,000 in the population, typically presenting with severe lower back pain, sciatica, genital sensory disturbance, and bladder or bowel dysfunction [1, 2]. It requires prompt diagnosis, appropriate investigations, and timely surgical decompression with postoperative rehabilitation to achieve a satisfactory outcome [2, 3]. Unfortunately, due to its rarity, it has become difficult to achieve a satisfactory outcome in a significant proportion of cases [2].

Cauda equina syndrome (CES) comes under two classifications, incomplete cauda equina syndrome (CES-I) and complete cauda equina syndrome with true retention (CES-R)

In CES-I, patients present with motor and sensory symptoms, including saddle anesthesia, but have yet to develop retention or incontinence of either bowel or bladder. At this point, immediate surgical intervention is needed, which gives the best chance of a full recovery. In CES-R, patients have already developed painless urinary retention and overflow incontinence. Decompression of the cauda equina nerves is still likely to be required, but there is a lesser chance of recovery of lost neurological function. It is vital to diagnose the condition before it deteriorates from CES-I to CES-R as the surgical outcome for patients with CES-I is generally favorable, whereas those with CES-R have a poorer prognosis, although around 70% of patients with CES-R have a socially acceptable long-term outcome [4].

More often than not, the outcome is already decided by the time of admission owing to a delay in presentation or referral, especially in low-resource settings. We present a tragic case of cauda equina syndrome with devastating long-term complications.

## Case presentation

A 55-year-old Sinhalese woman presented to the gynecology ward with a vulval abscess. She also complained of involuntary leakage of urine for the last 7 years. The incontinence was throughout both day and night. She did not have an urge to pass urine. The urinary flow was intermittent. There was no fecal incontinence. Sexual activity was also compromised due to coital incontinence.

Over the last 7 years, she had been admitted on three occasions for urinary tract infections to a gynecology ward at a tertiary hospital. The reason for pyelonephritis on the last admission was attributed to hydronephrosis. She had also been on clinic follow-up for urinary incontinence and had been treated with antimuscarinics and lifestyle modifications. She had not undergone any surgery for urinary incontinence. As there had not been any improvement, she had later defaulted follow-up.

On abdominal examination, a distended bladder was found. Neurological examination revealed a saddle sensory loss of S2–S4 dermatomes. There was no sensory loss over the lower limbs. Bladder sensation was absent, but there was some degree (reduced) of anal sphincter tone. Motor functions and reflexes were normal in the limbs.

**Fig. 1:**
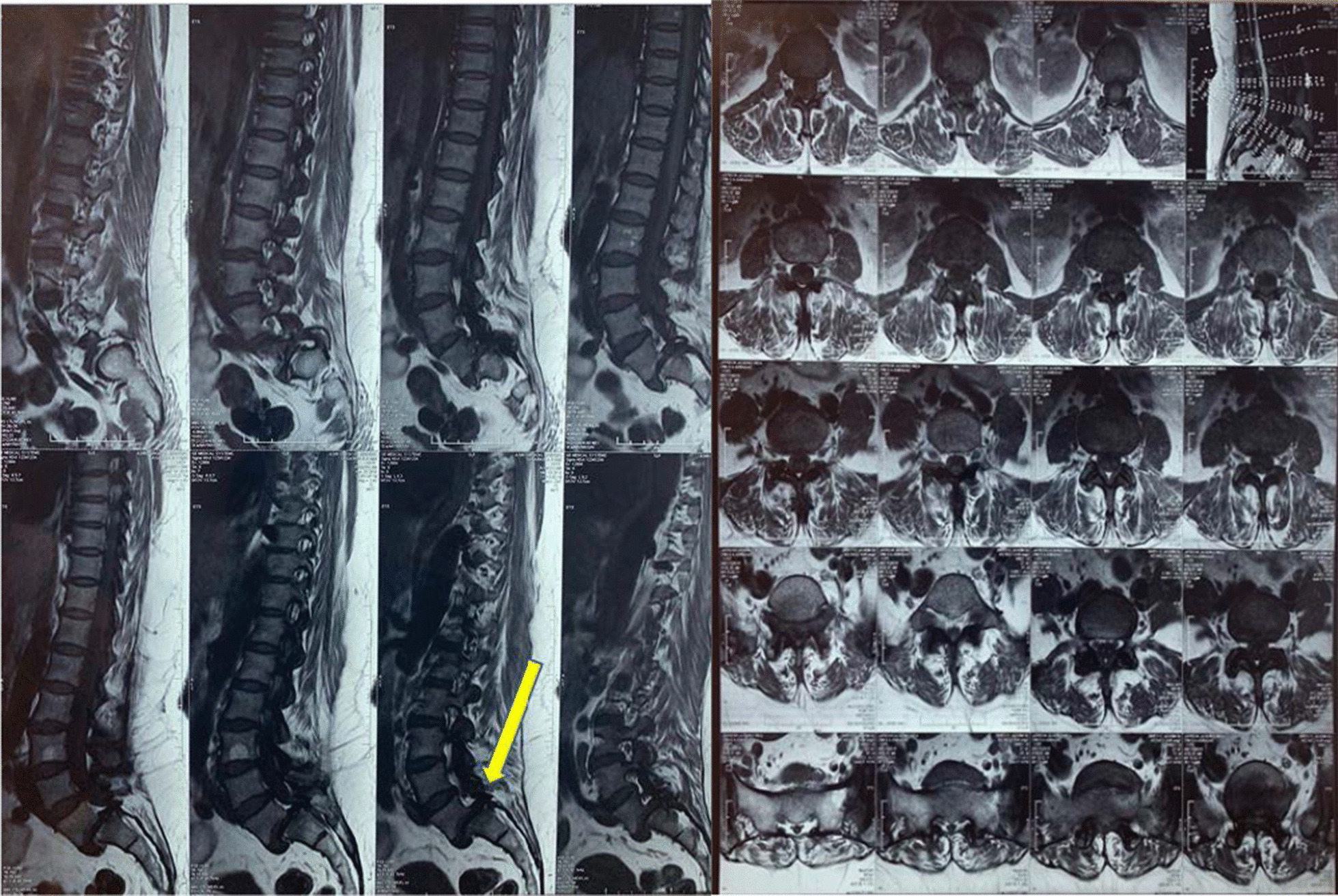
Sagittal and axial lumbosacral MRI showing L5–S1 spondylolisthesis. Arrow shows the site of spinal cord compression

Magnetic resonance imaging (MRI) revealed L5–S1 spondylolisthesis (Fig. 1). Computerized tomography (CT) of the brain was normal. Ultrasound imaging revealed a distended bladder with bilateral hydroureter and hydronephrosis.

An incision and drainage with concomitant intravenous antibiotics were started for the vulval abscess. An indwelling catheter was placed to decompress the distended bladder and to reduce vulval excoriations due to urine. Despite bladder decompression, serum creatinine remained elevated with persistent bilateral hydronephrosis indicative of secondary bilateral vesicoureteric junctional obstruction due to a grossly thickened bladder wall. Thus, bilateral ureteric stents were placed. Subsequently the serum creatinine improved from 6.54 to 2.57 mg/dL, and upper tract dilation reduced significantly.

## Discussion

This case illustrates the failure to recognize the presence of acute CES, which was likely due to an atypical presentation, low index of suspicion, and improper follow-up. This delay in appropriate therapy led to the catastrophic sequelae.

**Fig. 2:**
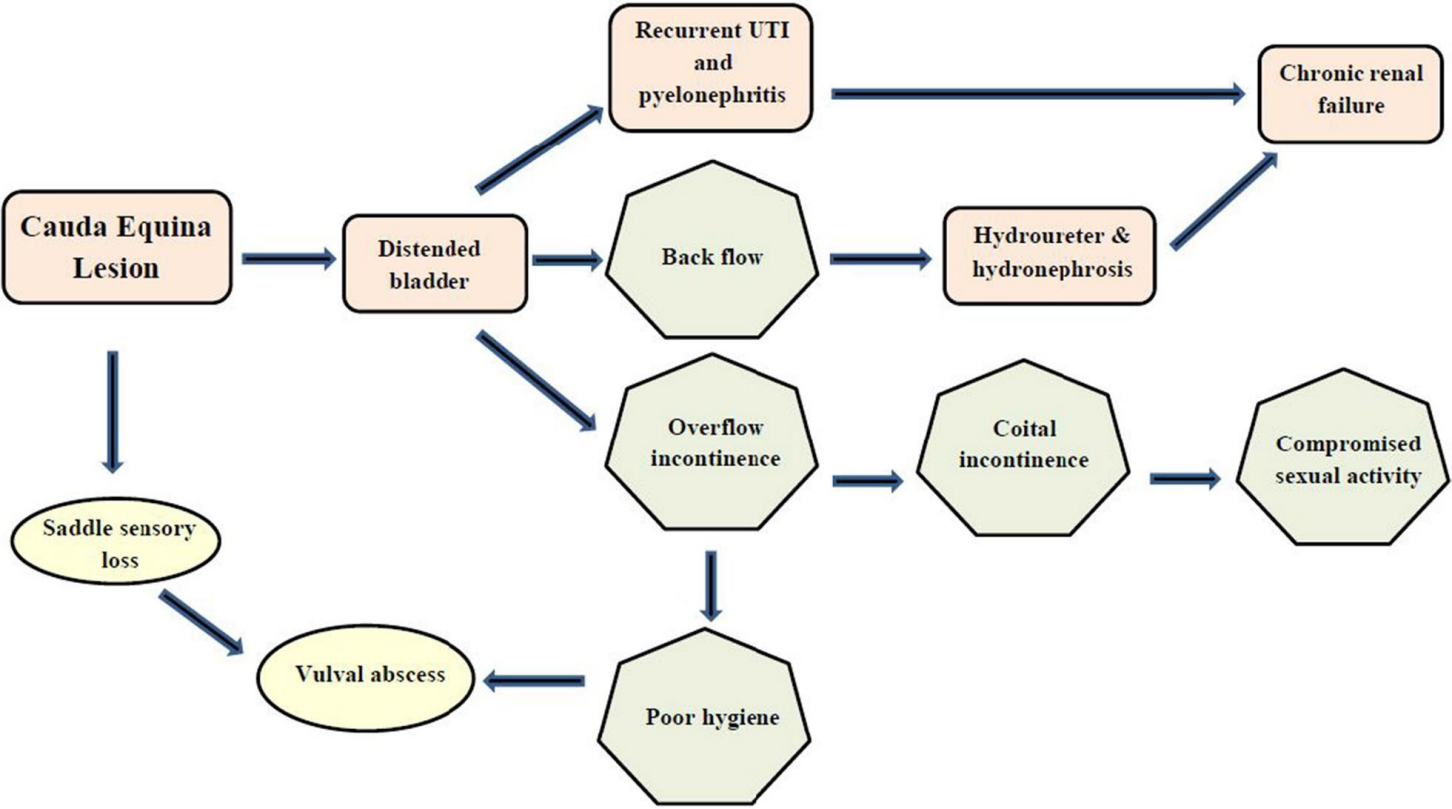
Flow chart to show the sequence of clinical events

**Fig. 3:**
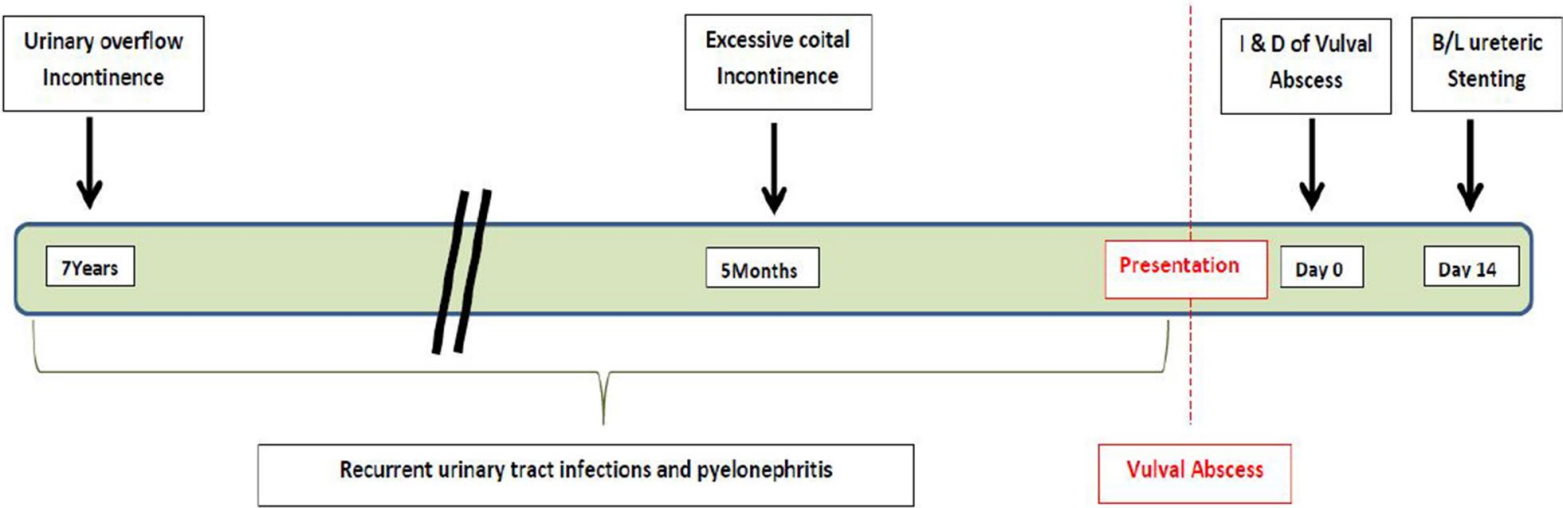
Timeline of events

Unfortunately, only one-fifth of the patients present with the classical triad of bilateral sciatica with lower limb weakness, saddle anesthesia, and sphincter disturbance [5]. In this patient, the atypical presentation of the loss of sensation in the perineum in the absence of sciatica and lower limb weakness probably accounted for the initial delay in presentation. During this admission, the vulval abscess prompted the detection of the saddle sensory loss that had not been picked up even by the patient. Thereafter, piecing together of the evidence of the clinical evaluation, that is, vulval abscess, saddle sensory loss, overflow incontinence, hydronephrosis, renal compromise, and MRI evidence, gave the complete picture (Fig. 2). The timeline of the development of complications is shown in Fig. 3.

Unfortunately, if the bladder dysfunction was properly evaluated during previous admissions, an overflow incontinence due to a neurogenic bladder would have been elicited by clinical evaluation alone. This would have, in turn, prompted a neurological examination that would have revealed a CES. A painless urine retention in CES leads to permanent bladder damage from irreversible stretching of the bladder wall resulting in a palpable bladder. Therefore, it is essential in a case of neurogenic bladder dysfunction to screen for CES, even if they do not present with classic symptoms [6]. Although this is basic undergraduate knowledge, it is common practice to look for urogynecological causes for incontinence rather than rare neurological causes. This compartmentalization of clinical features and the lack of a holistic approach to patient management is a result of involvement of multiple specialists in patient care. If catastrophes of this nature are to be prevented, actual discussion among clinicians and not mere referrals are needed, especially in the current concept of multidisciplinary care.

Diagnostic imaging with computerized tomography (CT) scan or magnetic resonance imaging (MRI) is mandatory in these cases. Surgical decompression, usually via laminectomy, is recommended if there is a disc herniation. It is generally accepted that CES is a surgical emergency as early decompression prevents irreversible neurological disability [2, 4, 5]. However, the lack of accurate imaging and prompt surgical decompression may be difficult to achieve in low-resource settings.

In summary, the triad of delayed presentation by the patient; physician-related factors such as not suspecting CES or referral to inappropriate speciality; and problems of low-resource settings such as delay or unavailability of magnetic resonance imaging (MRI) and inability to perform surgery due to a lack of beds or theater time in a tertiary care center will contribute to make an already bad situation worse [2]. It is probable that all of these factors contributed to this patient’s outcome.

## Conclusion

This is a case that demonstrates the tragic long-term sequelae of undiagnosed CES. It illustrates the importance of clinical suspicion and prompt referral to a neurosurgical unit to take timely action.

## Data Availability

Not applicable.
